# Assessing local stromal alterations in human ovarian cancer subtypes via second harmonic generation microscopy and analysis

**DOI:** 10.1117/1.JBO.22.11.116008

**Published:** 2017-11-29

**Authors:** Kirby R. Campbell, Paul J. Campagnola

**Affiliations:** University of Wisconsin–Madison, Department of Biomedical Engineering, Madison, Wisconsin, United States

**Keywords:** collagen, ovarian cancer, second harmonic generation

## Abstract

The collagen architecture in all human ovarian cancers is substantially remodeled, where these alterations are manifested in different fiber widths, fiber patterns, and fibril size and packing. Second harmonic generation (SHG) microscopy has differentiated normal tissues from high-grade serous (HGS) tumors with high accuracy; however, the classification between low-grade serous, endometrioid, and benign tumors was less successful. We postulate this is due to known higher genetic variation in these tissues relative to HGS tumors, which are genetically similar, and this results in more heterogeneous collagen remodeling in the respective matrix. Here, we examine fiber widths and SHG emission intensity and directionality locally within images (e.g., 10×10  microns) and show that normal tissues and HGS tumors are more uniform in fiber properties as well as in fibril size and packing than the other tissues. Moreover, these distributions are in good agreement with phase matching considerations relating SHG emission directionality and intensity. The findings show that in addition to average collagen assembly properties the intrinsic heterogeneity must also be considered as another aspect of characterization. These local analyses showed differences not shown in pure intensity-based image analyses and may provide further insight into disease etiology of the different tumor subtypes.

## 1 Introduction

Ovarian cancer remains the most lethal gynecologic malignancy mainly due to poor understanding of the early molecular and genetic origin and pathogenesis of ovarian carcinomas.^1^^,^^2^ Women diagnosed with ovarian cancer have an aggregate 5-year survival rate of ∼45%. However, the specific rates are highly dependent on the stage of the disease at the time of diagnosis. For example, disease localized to the ovary has a 5-year survival rate of ∼92%, but this sharply decreases to 27% for metastatic disease (American Cancer Society Facts and Figures 2016). Early detection is difficult due to vague symptoms (e.g., bloating and abdominal discomfort) and lack of effective clinical screening/imaging tests. For example, with currently available diagnostic imaging modalities, including positron emission tomography, computed tomography, magnetic resonance imaging, and ultrasound, only 15% of patients are diagnosed at stage I when the disease is localized to the ovary or fallopian tube.^3^^–^^6^ Similarly, the combination of the CA-125 tumor marker and transvaginal ultrasound has been investigated as screening strategies; however, the methods, even in combination, are not sufficiently selective or specific to be employed as clinical diagnostic tests for early detection.^3^^,^^7^ Superior imaging tools are needed for diagnostic/prognostic purposes and to better understand disease etiology.

Recently, clinicopathological observations and molecular genetic studies have identified several subtypes of ovarian tumors categorized under two genetically and pathologically distinct groups: types I and II.^8^^,^^9^ Type I tumors include borderline, mucinous, endometrioid cancers, and low-grade serous (LGS).^10^ High-grade serous (HGS) ovarian malignancies are classified as type II tumors and are the most common type of ovarian carcinoma comprising 70% of the total diagnoses.^9^ This new classification has elucidated the need for developing subtype-specific treatment strategies; however, no current clinically available diagnostic modality has the capability of adequately detecting and classifying the different subtypes.^11^^,^^12^ For example, p53 staining is a standard pathology tool, but it lacks sensitivity for distinguishing LGS and HGS tumors. An additional complication is that the type I tumors have a diverse set of associated proto-oncogene markers, including KRAS, BRA and ERBB2, and others.^13^ In contrast, most HGS tumors are genetically homogeneous, where essentially all are TP53 positive and many have associated BRCA I/II mutations.

Current pathological classifications for most cancers, including those of the ovary, are primarily based on cell phenotype and, to a lesser extent, genetic markers. However, the collagen in the extracellular matrix (ECM) is extensively remodeled in essentially all epithelial cancers,^14^^–^^20^ and imaging this structure offers the opportunity of providing a new, label-free biomarker. For example, we have previously characterized a spectrum of ovarian tumors as well as high risk and normal ovarian stromal tissues using second harmonic generation (SHG) imaging.^15^ This collagen specific and sensitive modality is ideal, as remodeling can present itself in the form of desmoplasia, modified morphology, and/or up-regulation of various collagen isoforms,^14^^,^^21^^–^^31^ all of which can be examined by SHG. Specifically, we have employed polarization sensitive approaches that probe molecular aspects,^28^^,^^30^^,^^32^^–^^35^ texture-based image analyses to probe fiber patterns,^15^^,^^36^^,^^37^ as well as the underlying SHG creation properties related to fibril size and packing to quantitatively differentiate ovarian tumors.^15^^,^^36^

Although these previous studies were collectively successful in classifying different tumors, especially in discerning HGS ovarian cancer, the lowest accuracies were in distinguishing between the various type I tumors.^14^^,^^15^ We postulate that this resulted from the highly averaged approach of analyzing collagen aspects over whole single optical sections, thereby omitting key microscopic information of tissue heterogeneity on smaller sizes than whole fields of view (e.g., 200 by 200  μm). In this paper, we extend our previous approaches to analyze collagen structure locally within the images to determine if local as well as global ECM alterations are present in different ovarian tissues and tumors. Specifically, by examining small regions (∼10×10  microns) within larger fields of view, we will examine if genetic heterogeneity within type I tumors is manifested by heterogeneity in the SHG contrast. To examine this possibility, we extract the collagen fiber/fiber bundle widths (and distributions therein) and the local distribution of the SHG emission direction ratio, $F_{\mathrm{SHG}}/B_{\mathrm{SHG}}$, which we have previously shown is a metric arising from fibril size and packing,^31^ to determine if ECM remodeling is heterogeneous and, moreover, if the heterogeneity varies between tissue types (normal and high risk tissues, benign, endometrioid, LGS and HGS tumors). This is an important consideration as this analysis will provide insight into disease etiology and progression of the different tumor subtypes. Additionally, we will correlate the SHG emission directionality with resulting SHG intensity to provide validation of previously developed theory.^31^

## 2 Methods

### 2.1 Tissue Removal and Preparation

All ovarian tissues were obtained using an institutional review board approved protocol from consented patients undergoing surgical debulking treatment for ovarian cancer or benign gynecological conditions. All tissues were immediately fixed in 4% formalin, refrigerated for 24 h, then switched to phosphate buffered saline. The specimens were sectioned in the collagen-rich areas near the surface epithelium using a Leica Vibratome 1200S (Leica Biosystems, Buffalo Grove, Illinois) to thicknesses of ∼100 to 150  μm. Tissues were classified by a gynecological pathologist for normal ovarian tissue (n=4), benign tumors (n=4), endometrioid type I (n=3), LGS type I (n=4 patient samples), and HGS type II (n=3 patient samples). We note the genomic mutation information for these tissues is unknown.

### 2.2 Second Harmonic Generation Imaging System

The essentials of the SHG imaging system have been described in detail previously by Chen et al.^25^ Briefly, a ∼100 femtosecond laser (Chameleon UltraTi:Sapphire, Santa Clara, California) was coupled to a home-built laser-scanning system on a fixed stage upright microscope (BX61WI, Olympus, Center Valley, Pennsylvania). A 40× 0.8 numerical aperture (NA) water immersion objective and a 0.9-NA condenser were used for excitation and collection, respectively, of the forward SHG signal. To equally probe all fiber orientations, circularly polarized light was used, and the purity was verified at the focus.^25^ The resulting image volumes consisted of lateral and axial resolutions of ∼0.7 and 2.5  μm, respectively. The backward SHG was collected in a nondescanned geometry, where the detector was in the infinity space. Both detectors were H7422-40P GaAsP photon counting photomultiplier tubes (Hamamatsu, Hamamatsu, Japan). The laser excitation and collected SHG wavelengths were 988 and 494 nm, respectively, and chosen based on the highest differentiation of the two sample groups in our previous study.^14^ The SHG signal was isolated with 20-nm bandpass filters centered at 494 nm (Semrock, Rochester, New York). Calibration of the forward (F) and backward (B) detection pathways was performed using the two-photon-excited fluorescence imaging of microspheres (Fluoresbrite YG 6.0  μm, Polysciences, Inc, Warrington, Pennsylvania) mounted in 1% porcine gelatin on similar glass slides and coverslip as the ovarian tissue slices. The F/B ratio of the collected isotropic emission of these microspheres is used to baseline the photon collection efficiency of the detection paths at the same 494-nm wavelength as the collected SHG signal used for the F/B analysis.

Simultaneous forward and backward SHG images were obtained every 1  μm through the entire depth of tissues in three distinct fields of view per patient. Images were acquired at two times digital zoom with a field-of-view of 170  μm by 170  μm with a field size of 512 by 512 pixels to sample at the Nyquist frequency. The first and last 10 optical sections of the image stacks were removed for boundary effect reasons leaving the remaining portion of the image stack (∼80 to 130  μm) to be used for analyses. Image stacks were first denoised and saturated pixels were removed using an intensity threshold based on stringent conditions for both forward and backward image stacks. For local analysis, individual pixel areas were intensity averaged for both the forward and backward channel responses. Three-dimensional (3-D) renderings were performed in Imaris (Bitplane AG, Zurich, Switzerland).

### 2.3 Monte Carlo Simulations of Second Harmonic Generation Directional Response

The measured F/B versus axial depth response is a coupled effect of initial SHG directional emission, denoted as $F_{\mathrm{SHG}}/B_{\mathrm{SHG}}$, and the subsequent SHG transport through the tissue, which is based largely on the reduced scattering coefficient, $\mu_{s}^{\prime }$, at 494 nm (absorption was considered negligible, as $\mu_{a}\ll \mu_{s}$, previously confirmed in ovarian tissue). The SHG directional emission ratios, $F_{\mathrm{SHG}}/B_{\mathrm{SHG}}$, were decoupled from the depth-dependent SHG response curves using Monte Carlo simulations based on an adapted Monte Carlo multilayer^38^ framework.^39^^,^^40^ Using parallel computing at the Center for High Throughput Computing at the University of Wisconsin–Madison, a series of forward simulations of the forward/backward emission profile was modeled (using all optical and geometrical specifics from our imaging system) versus depth of each optical section based on the corresponding measured optical properties ($\mu_{s}^{\prime }$ and refractive index, as measured previously)^14^ of each tissue section and initial guesses of the emission directionality ($F_{\mathrm{SHG}}/B_{\mathrm{SHG}}$ ratios of 1 to 20 in 0.3 increments). The output data from the simulations were stored in tables, permitting all the image processing to be performed in a custom MATLAB program (code available upon request).

### 2.4 Statistical Analyses

Pearson correlation coefficients were calculated to statistically evaluate similarity of the extracted $F_{\mathrm{SHG}}/B_{\mathrm{SHG}}$ creation ratio matrices to the corresponding SHG intensity grayscale images for all sample image volumes. Values range from 1.0 for two matrices that have a perfect linear relationship and −1.0 for a perfect inverse relationship, with 0.0 representing complete uncorrelation. For equal comparisons, local 30×30-pixel regions of $F_{\mathrm{SHG}}/B_{\mathrm{SHG}}$ creation ratio matrices (area optimized in Sec. 3) and their respective SHG images were each self-normalized for every optical section in the Pearson correlation coefficient calculations. Finally, two-sample t-tests were performed on the averages of the Pearson correlation coefficients of each image stack for all tissue groups using the statistics toolbox in Origin 9.1 (OriginLab, Northampton, Massachusetts).

## 3 Results

### 3.1 Collagen Fiber Morphology

**Fig. 1:**
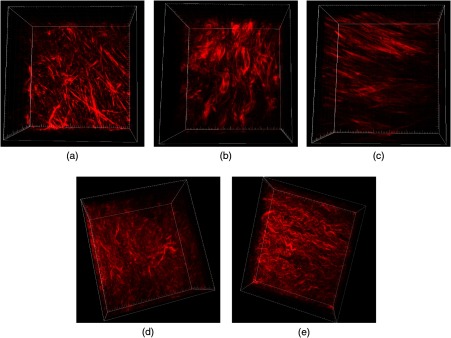
Representative 3-D renderings of forward-directed SHG images of (a) normal, (b) benign tumor, (c) endometrioid, (d) low-grade, and (e) high-grade serous ovarian tumors obtained at 988-nm excitation. Field size=170  μm×170  μm.

The left to right images in Fig. 1 show representative 3-D SHG renderings of normal, benign, endometrioid, LGS, and HGS ovarian cancer, respectively. Normal ovarian tissue contains loose, mesh-like collagen fibers, whereas benign tumors are highly fibrotic with complex networks of large, thick collagen fibers and bundles of overlapping fibers. The malignant type I and II tumors have vastly different collagen morphologies depending on the sample group. Endometrioid and LGS tissue samples are more heterogeneous in nature, but in general, LGS tissues are highly fibrotic, consisting of a tightly packed matrix of shorter collagen fibers, whereas endometrioid tumors have sparser but longer, straight-aligned collagen fibers. In contrast, HGS tissue morphology is highly conserved within the patient population and tissues typically display densely packed, aligned fibers often appearing wavy in nature. Different sampling regions did not show significant variations in the collagen coverage in each tissue type; moreover, the analyses are limited to the collagen dense tunica albuginea areas near the surface epithelium.

**Fig. 2:**
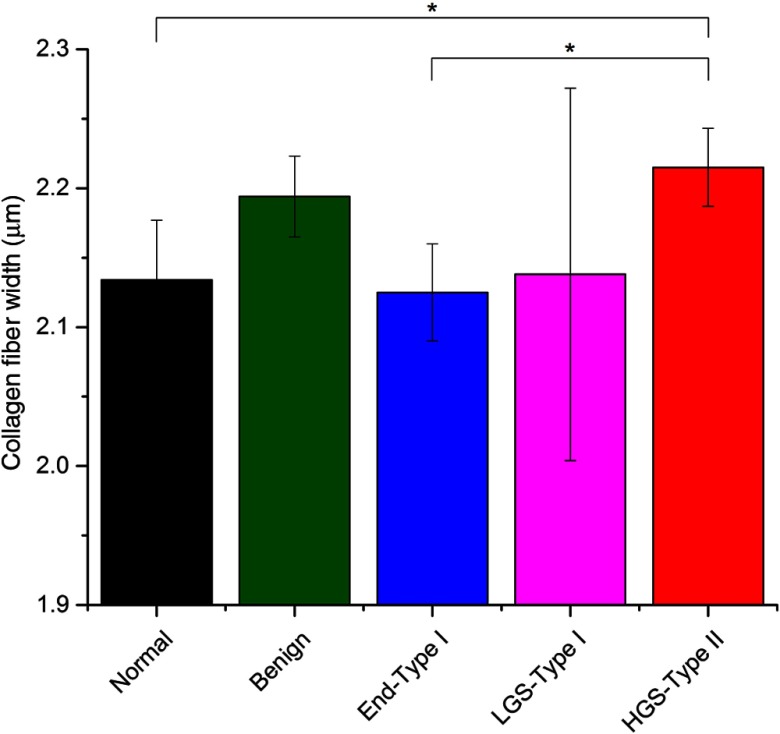
Average collagen fiber widths for normal (black), benign tumor tissue (green), endometrioid-type I (blue), low-grade (magenta), and high-grade (red) serous ovarian cancer quantified by CT-Fire software. Error bars depict standard error.

We used CT-FIRE (standalone MATLAB program, Ref. 41) to quantify average fiber widths and their distribution in the data sets. The CT-FIRE program utilizes curvelet transform in conjunction with a fiber extraction algorithm for extracting descriptive fiber statistics in each image. In many instances, this fiber detection algorithm is unable to decipher individual fibers versus fiber bundles. The average extracted fiber/fiber bundle width values (standard error in parentheses) were (i) normal tissue: 2.13(0.04)  μm, (ii) benign tumor: 2.19(0.03)  μm, (iii) endometrioid 2.13(0.04)  μm, (iv) LGS: 2.14(0.13)  μm, and (v) HGS: 2.2(0.03)  μm (see Fig. 2). Using this analysis, the fiber/fiber bundles in the HGS tissues were thicker and significantly different from the normal and endometrioid tissues; however, the high degree of heterogeneity of the fiber widths extracted from the LGS tissues resulted in no significant difference among LGS and HGS. More importantly, this shows the large heterogeneity in the widths of the LGS tissues compared with all the other groups, suggesting that the etiology and progression is different from the other classes.

### 3.2 Local Second Harmonic Generation Directional Measurements

#### 3.2.1 Optimization of parameters

**Fig. 3:**
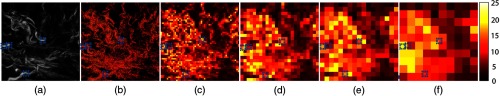
(a) Representative LGS SHG image and local $F_{\mathrm{SHG}}/B_{\mathrm{SHG}}$ heat maps for (b) single pixel fits, (c) 10×10, (d) 20×20, and (e) 30×30, and (f) 50×50-pixel patch fits. The 30×30 grids fully incorporate fiber bundle structures, as outlined by blue dashed boxes.

We pursue this issue of heterogeneity further by extracting the SHG $F_{\mathrm{SHG}}/B_{\mathrm{SHG}}$ emission ratios locally in different regions of the image and determine if the distribution in values is different between classes and further determine its correlation with the resulting SHG intensity. We have previously shown that the SHG emission directionality arises from the fibril size and packing and is related to intensity by phase-matching considerations.^31^ We previously extracted the emission directionality as a metric to compare different ovarian tissues using a frame-averaged approach,^14^ which is not sensitive to variations within the images. For local assessment, we first needed to optimize the area size to be analyzed. Initially, we explored a pixel-by-pixel-based approach, but Poisson noise prohibited accurate fits for every pixel. We then ran a series of optimization tests on an LGS sample to discern the best pixel patch dimension by starting with 500×500  pixels and dividing into 10×10, 20×20, 30×30, and 50×50 pixel patches (see Fig. 3). Highlighted by the blue boxes in Fig. 3, the 10×10 and 20×20 patches were too small to incorporate fiber and fiber bundle structures in their entirety, whereas the 30-×30-pixel patches analysis successfully incorporated full fiber structures and is used for the following analysis. The larger 50×50 averaged too many pixels to the degree that many individual fibers were being averaged and specific fiber information was lost. Larger patches will converge to the frame-averaged approach.

#### 3.2.2 Extraction of local second harmonic generation emission directionality

**Fig. 4:**
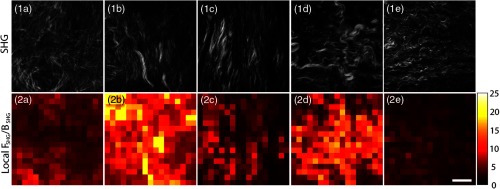
Representative (row 1) SHG images and (row 2) corresponding $F_{\mathrm{SHG}}/B_{\mathrm{SHG}}$ patch-wise heat maps for (column a) normal, (column b) benign tumor, (column c) endometrioid, (columns d) LGS, and (column e) HGS ovarian tissue. Scale bar=35  μm.

We next extracted the mean local SHG emission $F_{\mathrm{SHG}}/B_{\mathrm{SHG}}$ ratio and the standard deviations within the entirety of each tissue volume image stack from the five different tissue classes using the Monte Carlo simulations described in Sec. 2. Figure 4 shows representative SHG images (top row) of normal tissue (column a), benign tumor (column b), endometrioid/type I (column c), LGS/type I (column d), and HGS/type II (column e) and their corresponding locally extracted $F_{\mathrm{SHG}}/B_{\mathrm{SHG}}$ values in the form of heatmaps (bottom row). In general, the benign tumor and LGS samples had higher overall $F_{\mathrm{SHG}}/B_{\mathrm{SHG}}$ values as was found in previous studies.^14^ In contrast, normal, endometrioid, and HGS samples showed much lower $F_{\mathrm{SHG}}/B_{\mathrm{SHG}}$ creation ratios, also shown in our previous work.^14^^,^^24^ Additionally, these local values were consistent across each tissue section of the patient group. For example, the most heterogeneous region of the normal and HGS patient group $F_{\mathrm{SHG}}/B_{\mathrm{SHG}}$ heat maps is shown in Figs. 4(2a) and 4(2e) revealing the uniformity of these two patient groups. In contrast, the LGS and benign tumors have higher degrees of heterogeneity within individual optical sections.

Overall, these respective local analyses trend with previous data for each sample groups.^14^ However, when taking a more in-depth examination of the local $F_{\mathrm{SHG}}/B_{\mathrm{SHG}}$ heat maps to the intensity of SHG contrast of individual fibers and smaller fiber bundles, many localized regions of the benign and LGS samples had remarkably higher $F_{\mathrm{SHG}}/B_{\mathrm{SHG}}$ values (∼15 to 20) than the average values. Examples of this are shown in the upper-left region of Fig. 4(2b) and throughout the LGS image section shown in Fig. 4(2d). We conclude these arise from locally thick, dense groups of fibers or fiber bundles. We quantify the heterogeneity within each group by analysis of the distribution of these values throughout the data sets.

The averaged respective values and their distributions extracted for each entire patient group are summarized in Table 1. Row 1 contains the average $F_{\mathrm{SHG}}/B_{\mathrm{SHG}}$ creation ratios with standard errors for each patient group averaging all the $F_{\mathrm{SHG}}/B_{\mathrm{SHG}}$ patch values throughout the tissue volume, and standard deviation data in row 2 quantifies the variability of extracted $F_{\mathrm{SHG}}/B_{\mathrm{SHG}}$ values throughout individual tissue volumes. By this account, benign and LGS tissue exemplify the largest range of extracted $F_{\mathrm{SHG}}/B_{\mathrm{SHG}}$ values within a certain image stack (i.e., most heterogeneous $F_{\mathrm{SHG}}/B_{\mathrm{SHG}}$ profile); whereas, endometrioid and HGS cancer tissues have the narrowest range of $F_{\mathrm{SHG}}/B_{\mathrm{SHG}}$ values (i.e., least heterogenous $F_{\mathrm{SHG}}/B_{\mathrm{SHG}}$ profile).

**Table 1:** Analyses of locally derived SHG emission ratios and corresponding distributions.

	Normal	Benign	Low grade endometrioid—type I	LGS—type I	HGS—type II
Mean $F_{\mathrm{SHG}}/B_{\mathrm{SHG}}$	4.0±0.3	8.8±0.6	3.0±0.2	5.1±0.6	2.7±0.2
Individual tissue $F_{\mathrm{SHG}}/B_{\mathrm{SHG}}$ Std. Dev.	2.0±0.4	3.9±0.4	1.7±0.2	3.5±0.5	1.7±0.3

We also note that the LGS and HGS sample sets have a trending relationship of extracted fiber widths to the standard deviations of extracted $F_{\mathrm{SHG}}/B_{\mathrm{SHG}}$ emission ratios. For example, the LGS tissues have much higher variances of extracted values of $F_{\mathrm{SHG}}/B_{\mathrm{SHG}}$ components and fiber widths when compared with the HGS tissue samples, which are more uniform in both metrics. Importantly, these tumors are not usually distinguishable by p53 staining but are easily discerned here.

**Fig. 5:**
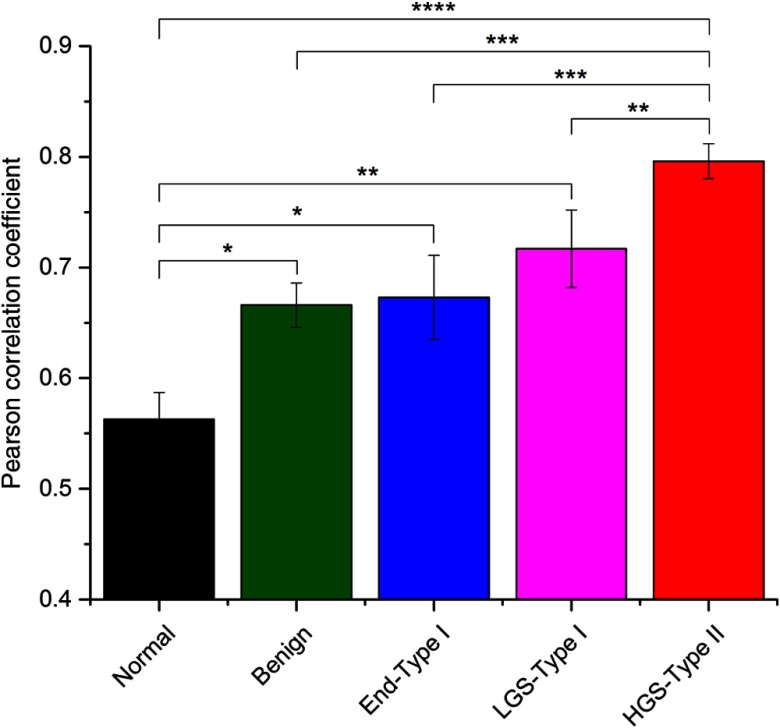
Average Pearson correlation coefficients evaluating colocalization of extracted local $F_{\mathrm{SHG}}/B_{\mathrm{SHG}}$ emission ratio matrices to corresponding SHG intensity images for normal (black), benign tumor tissue (green), endometrioid-type I (blue), low-grade (magenta), and high-grade (red) serous ovarian cancer samples. Error bars depict standard error.

Next, correlation analysis was performed to statistically evaluate similarity of the locally extracted $F_{\mathrm{SHG}}/B_{\mathrm{SHG}}$ values to the corresponding SHG intensity grayscale images. In the simplest framework, these are related to phase-matching considerations, where smaller phase mismatch, Δk, results in brighter SHG (see Sec. 4) and more forward-directed emission. However, the phase mismatch is not necessarily single valued in biological tissues due to structure heterogeneity, which we are demonstrating is different between the tissue classes. The total coefficients for all optical sections were averaged for the five tissues and are shown in Fig. 5. By this metric, normal tissue had significantly lower Pearson correlation coefficients (0.56±0.02) compared with benign tumors (0.66±0.02), endometrioid (0.67±0.04), LGS (0.71±0.04), and HGS (0.80±0.02), respectively.

In contrast, HGS tissues had significantly higher Pearson correlation coefficients than the other four sample sets, indicating high uniformity of fibril size and low distribution of Δk values. The lower degree of colocalization of normal, benign, endometrioid, and LGS tissues compared with HGS indicates these tissues have higher distributions of fibril/domain sizes (i.e., higher degree of heterogeneity in fiber architecture) resulting in more extreme values $F_{\mathrm{SHG}}/B_{\mathrm{SHG}}$ emission ratios compared with respective SHG intensities, i.e., there is a larger distribution of Δk values. We note that this analysis provides a clearer picture of the tissue than simple intensity analysis. For example, we found essentially no discrimination between the tissues using gray-level co-occurrence matrix (GLCM) readouts of energy, entropy, and heterogeneity. This suggests that detailed SHG analysis of the underlying contrast based on phase mismatch regionally is important relative to either frame-by-frame averaging or GLCM nearest neighbor analysis.

## 4 Discussion

This work allows local examination of fibrillar aspects in SHG mages, where intrinsic heterogeneity may be useful in classifying diseased tissues, e.g., different ovarian cancer subtypes have different uniformities in structure. This may have the most impact in differentiating tissues, where specific optical markers are yet to be established, e.g., between LGS and HGS tumors. We draw upon a previously developed heuristic model for SHG creation emission direction based on relaxed phase-matching conditions, which account for dispersion, randomness, and axial contributions from the media.^31^ These nonideal conditions give rise to a distribution of forward and backward emitted photons to conserve momentum. This phase mismatch also impacts the SHG intensity, where smaller mismatches result in brighter and more forward directed SHG signals and is given by

$$E_{2\omega }=\kappa E_{\omega }^{2}\frac{\sin(\Delta kL/2)}{\Delta kL/2}.$$ (1)

Spatially resolved local analysis of $F_{\mathrm{SHG}}/B_{\mathrm{SHG}}$ and correlation with SHG intensity provided direct validation of the theory as well as comparison of various tissues, where the collagen remodeling is different. For example, tissues with larger heterogeneity in structure, i.e., the LGS will have the largest distribution of Δk values and larger resulting distribution of $F_{\mathrm{SHG}}/B_{\mathrm{SHG}}$ values. The HGS tissues had the highest Pearson correlation coefficients (∼0.8) between $F_{\mathrm{SHG}}/B_{\mathrm{SHG}}$ and SHG intensity, where this would imply a narrow distribution of Δk values and domain sizes relative to $\lambda_{\mathrm{SHG}}$. This is consistent with the narrow distribution of smaller fibrils (∼60  nm) we had reported previously through TEM analysis.^24^ Moreover, this also borne out in the narrow distribution of fiber sizes from the image data (Fig. 2). The significantly lower Pearson correlation coefficients between emission directionality and intensity of the normal, benign, endometrioid, and LGS samples are indicative of larger distributions of fibril diameters and their packing. Specifically, many regions of the collagen in these sample groups exhibited high and low values of $F_{\mathrm{SHG}}/B_{\mathrm{SHG}}$ ratios regardless of the SHG intensities of these regions. This is likely due to heterogeneities in the collagen fibril sizes, larger single fibrils, or overlapping smaller fibrils (forming a larger effective domain) similar in size to $\lambda_{\mathrm{SHG}}$.

We note that we observed a related effect in our efforts in classification using texture analysis,^15^ where we found that the differentiation of these type I tissues was lower than either the normal tissues or HGS tumors. We had found the lowest accuracy between LGS tumors and benign tumors, where here we found the largest variations in the SHG metrics in these groups. Interestingly, there have been suggestions that the benign tumors can be precursors to LGS tumors.^9^ These collective findings further indicate that not only are the magnitude of the SHG metrics important, but the heterogeneity within also needs to be considered for more optimal characterization and classification. Notably, intensity nearest neighbor metrics based on GLCM showed no differences between the tissues, which showed strong differences of SHG attributes using local analyses. We further suggest these findings are related to the genetic variations between type I and type II tumors.

Although this technique relies on the assumption of a constant reduced scattering coefficient measured by bulk tissue-averaged measurements, we note these tissues are several scattering lengths thick, and while collagen morphology will vary within each tissue, local varying SHG emission will experience the same average scattering properties for subsequent photon propagation. We further note that we typically measure average bulk properties in different regions of tissues and find little variation site to site.

While the current work was limited to ovarian cancer, this overall approach could be important for other diseases that have less uniformity in tissue structure. For example, the ovary is predominantly comprised of collagen near the surface epithelium, whereas this is not necessarily the case in other tissues such as breast cancer, which is composed of other components in addition to collagen (e.g., fat and other cellular compartments). Indeed, frame-averaged SHG directional analysis of breast cancer tissues has not produced consistent results (unpublished data). This consideration is also operative in many fibroses, e.g., those of the lung, liver, and kidney. For extensions to other such tissues, the optimal size would need to be matched to both the collagen density and fiber lengths. In the limit of dense collagen (e.g., over 70% coverage), as in the case of ovarian stroma, the relevant parameter is mainly the fiber length, so the grid size was chosen such that the full fiber lengths were matched.

## 5 Conclusions

Our results here show that quantifying the intrinsic heterogeneity of fibril/fiber architecture between different ovarian tumors is another key to having accurate characterization tools. Specifically, the ability to distinguish ovarian tissues based on local, detailed analyses of the collagen ECM is important to increase accuracy of clinical classification and improve our understanding of the etiologies of different types of ovarian cancers. We found that the SHG metrics extracted from type II tumors are homogeneous by comparison with type I and is consistent with their respective genetic profiles. Importantly, the $F_{\mathrm{SHG}}/B_{\mathrm{SHG}}$ analysis probes subresolution assembly on the microscope without the need for difficult TEM preparation and analysis. Furthermore, this analysis may also be correlated with genomic analytics to provide a full diagnostic assessment and potentially facilitate in the development of specific chemotherapeutics to ovarian cancer type.
